# Bacterial association and comparison between lung and intestine in rats

**DOI:** 10.1042/BSR20191570

**Published:** 2020-04-29

**Authors:** Tian-hao Liu, Chen-yang Zhang, Ahmad Ud Din, Ning Li, Qian Wang, Jing-ze Yu, Zhen-yuan Xu, Chen-xi Li, Xiao-mei Zhang, Jia-li Yuan, Li-guo Chen, Zhong-shan Yang

**Affiliations:** 1 College of Chinese medicine, Jinan University, Guangzhou, Guangdong, China; 2 Yunnan Key Laboratory of Molecular Biology of Chinese Medicine, Yunnan University of Chinese Medicine, Kunming, Yunnan, China; 3 College of Chinese Medicine, Hunan University of traditional Chinese Medicine, Changsha, Hunan, China; 4 Drug Discovery Research Center, Southwest Medical University, Luzhou, Sichuan, China; 5 Yan’an Hospital Affiliated to Kunming Medical University, Key Laboratory of Cardiovascular Disease of Yunnan Province, Kunming, Yunnan, China; 6 School of Finance, Yunnan University of Finance and Economics, Kunming, Yunnan, China

**Keywords:** 16S rRNA, Bacteria, Community Structure and Function, Intestine, Lung

## Abstract

The association between lung and intestine has already been reported, but the differences in community structures or functions between lung and intestine bacteria yet need to explore. To explore the differences in community structures or functions, the lung tissues and fecal contents in rats were collected and analyzed through 16S rRNA sequencing. It was found that intestine bacteria was more abundant and diverse than lung bacteria. In intestine bacteria, Firmicutes and Bacteroides were identified as major phyla while *Lactobacillus* was among the most abundant genus. However, in lung the major identified phylum was *Proteobacteria* and genus *Pseudomonas* was most prominent genus. On the other hand, in contrast the lung bacteria was more concentrated in cytoskeleton and function in energy production and conversion. While, intestine bacteria were enriched in RNA processing, modification chromatin structure, dynamics and amino acid metabolism. The study provides the basis for understanding the relationships between lung and intestine bacteria.

## Introduction

Over the last few decades, the influence of gut microbiota on lung immune responses and physiology is emerging as links between bacteria “gut-lung axis”, through the underlying mechanisms [1]. Intestine bacteria play an important role in regulating immune function and promoting metabolism [2–4] and is closely related to respiratory diseases such as asthma, chronic obstructive pulmonary disease (COPD) etc. [5–7]. Intestine microbiota or its metabolites in certain host cells express the histidine decarboxylase enzyme (HDC), a pyridoxal-5′-phosphate (PLP)-dependent enzyme, which catalyzes the decarboxylation of histidine to histamine, and can have immunological consequences at distant mucosal sites within the lung [8]. Microbiota-derived short-chain fatty acids (SCFAs) are another major metabolites that function in regulating the intestinal barrier and enhancing the immunity [9,10]. SCFAs play an important role in various diseases, such as (chronic obstructive pulmonary disease) COPD and asthma [11–13]. Dysbiosis in intestine bacteria can exacerbate lung inflammation through T helper type 2 cell (Th2), CD4 T cells responses, and SCFAs ameliorates enhanced lung inflammation susceptibility by modulating the activity of T cells, dendritic cells (DCs) and expression of Th1-associated factors [14,15]. Additionally, the theory of traditional Chinese medicine (TCM) on the relationship between the lung and the large intestine suggests the functional correlation between lung and intestine [16–18]. When acupuncture and moxibustion are adopted to treat coliform diseases or respiratory diseases at large intestine and lung meridians, related researches showed that intestinal diseases could lead to inflammatory immune cells in the lung [19]. These increasing evidences support the correlation between lung and intestine. Therefore, we believe that the lung bacteria could be correlated with intestine bacteria.

**Figure 1:**
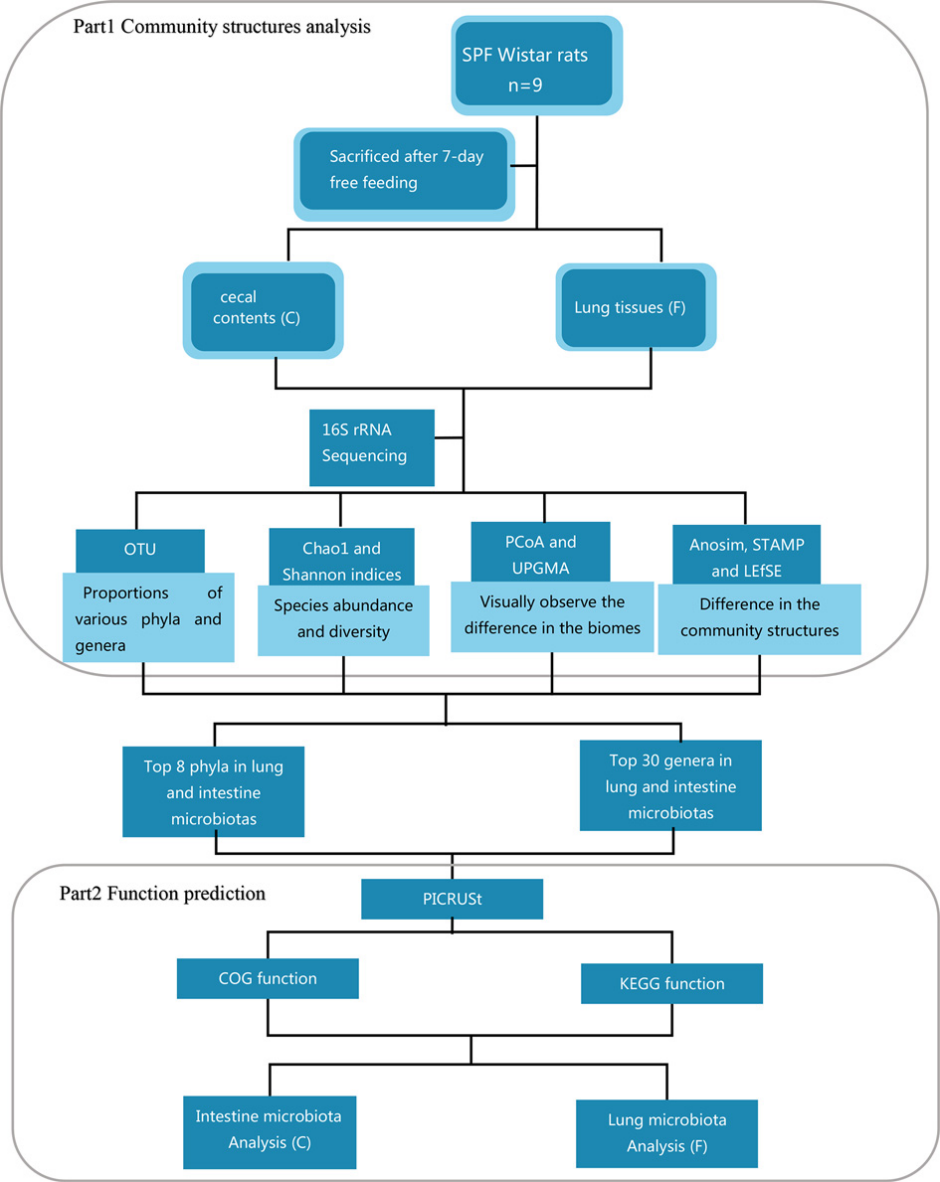
The flowchart reveals comparison of the community structures and functions between lung and intestine bacteria in rats

In the study, we chose lung and intestine bacteria as the research objects. The pulmonary bacteria in the context of human pulmonary health is mainly associated with respiratory diseases [20–22]. For example, *Pseudomonas aeruginosa* can cause chronic respiratory infections in the lungs [23–25]. Therefore, human body need to maintain an optimum level of bacteria. Intestine microbiota has been widely explored than lung bacteria. However, the comparison of structures and functions of lung and gut bacteria has rarely been reported. The co-relation between lung and intestine bacteria or the differences in the community structures and their functions still need high degree of accuracy to be explore. The present study explored lung and intestine bacteria via the 16S rRNA sequencing technology. After 7 days, multiple samples from lung tissues (F) and cecal contents (C) were collected for the sequence analysis and then the function analysis was conducted by Kyoto Encyclopedia of Genes and Genomes (KEGG) and Cluster of Orthologous Group (COG). Finally, we compared the structures and functions, and discussed reciprocal connections between lung and intestine bacteria (Figure 1).

## Methods

### Rats and samples

Nine SPF Wistar rats (Chengdu Dashuo Experimental Animal Co., Ltd.) were used and study was conducted according to the relevant guidelines and regulations. The samples were gathered from 9 rats of approximately 5- to 6-week-old male. The rats were freely fed with rats (Beijing Huafukang Biotechnology Co., Ltd.) and water for 1 week in standard environmental conditions in animal experiment center of Yunnan University of Chinese Medicine. After 7 days of feeding, the rats were performed under sodium pentobarbital anesthesia and then killed by neck removal to make to minimize suffering. Then, the cecal contents and lung tissues of each rat were collected into 10-ml centrifuge tubes, respectively, designated as “C” and “F” and then stored at −80°C for further analysis.

### DNA extraction and 16S rRNA sequencing

DNA was extracted and purified using Genome Extraction Kit of Tiangen Soil (P5103) and Qubit ds DNA BR Assay Kit (1751577) and Qubit ds DNA HS Assay Kit (1828332) following the manufacture’s protocols. Then, a high-throughput sequencing library was constructed by GENEWIZ (Suzhou, China) via Illumina MiSeq platform. DNA samples were quantified using a Qubit 2.0 Fluorometer (Invitrogen, Carlsbad, CA). Using 30–50 ng microbial genomic DNA as the template, a series of PCR primers (5′-CCTACGGRRBGCASCAGKVRVGAAT-3′; 5′-GGACTACNVGGGTWTCTAATCC-3′) designed with GENEWIZ were used to amplify the V3-V4 hypervariable region of the prokaryotic 16S rRNA gene. In addition, the operation process refered to literature [26,27]. The sequence data are available at the NIH Sequence Read Archive (https://submit.ncbi.nlm.nih.gov/subs/bioproject/) under the Bioproject accession number PRJNA496360.

### Statistical analysis

The 16S rRNA data analysis was performed in QIIME package (1.9.1) and R software (2.15.3). The forward and reverse reads obtained by double-end sequencing were first connected in pairs, followed by filtering the sequences containing N in the splicing result and retaining the sequence with a length greater than 200 bp. After mass filtration, the chimeric sequences were removed, and the resulting sequences were used for the analysis of Operational Taxonomic Units (OTU). Sequence clustering was performed with VSEARCH (1.9.6) (sequence similarity was set to 97%) and aligned with 16S rRNA reference database Silva 119. The Ribosomal Database Program (RDP) classifier Bayesian algorithm was used to perform the species taxonomic analysis with the representative sequence of OTU. The community composition of each sample was counted at different classification levels.

Based on the OTU analysis results, the samples were randomly selected. Then, the relative abundances at the phylum and genus levels, Shannon, and Chao1 indices were calculated using QIIME (1.9.1) [28–30]. The two groups were clustered based on the Bray–Curtis distance matrix, followed by principal co-ordinates analysis (PCoA) and unweighted pair group method with arithmetic mean (UPGMA) using R software (2.15.3) [31–34]. To further investigate the differences in the community structures between lung and intestine bacteria, Anosim analysis was performed via R software (2.15.3) [35,36]. To explore significant differences in the abundance of the genera, STAMP analysis was performed using STAMP software (v2.1.3) [37,38]. LDA Effect Size (LEfSe) analysis was performed by the online LEfSe analysis tool for differential microbiome analysis (http://huttenhower.sph.harvard.edu/galaxy/root?tool_id=lefse_upload) [39,40]. To correlate lung and intestine microbiota, analysis was performed using PICRUSt, including COG and KEGG function analysis [41,42]. The comparison between the two groups was conducted using *t*-test. *P* value at 0.05 was considered to be statistically significant.

## Results

### General structural characteristics of lung and intestine bacteria

The original reads had an average length of 300 bp and were optimized for sequencing the data (Supplementary Table S1). The number of OTUs in lung microbiota obtained was 389, whereas 320 in intestine bacteria. Collectively, there are 266 common OTUs detected between the two groups, including 123 unique OTUs in lung bacteria and 54 unique OTUs in intestine. The number of OTUs in lung is 127% more than that in intestine bacteria.

**Figure 2:**
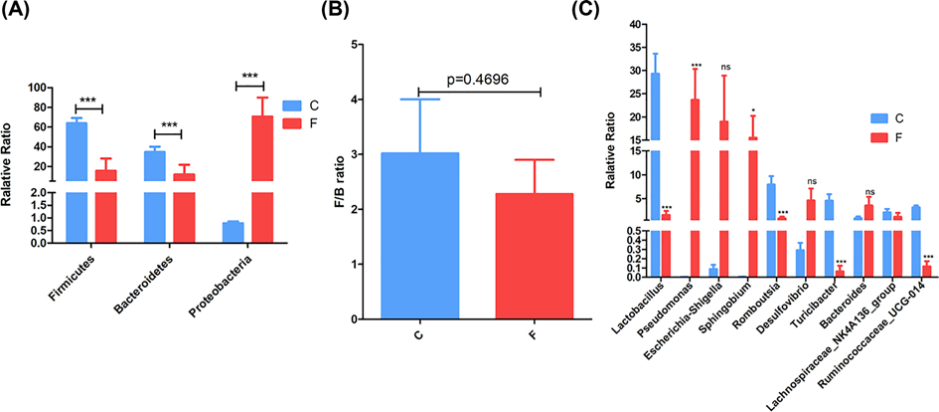
General structural characteristics of lung and intestine (F: lung, C: intestine) bacteria (**A**) Different relative abundance at the phylum level. (**B**) F/B ratio. (**C**) Different relative abundance at the genus level. The results were showed as mean ± sem. Statistical significance was denoted by *P<0.05; ***P<0.001.

Furthermore, phyla level results show that dominant phyla in the two anatomical sites were Firmicutes, Proteobacteria and Bacteroidetes, followed by less abundant phyla. Firmicutes and Bacteroidetes in lung bacteria were significantly less than those in intestine bacteria, whereas Proteobacteria in lung bacteria was significantly more than that in intestine bacteria (*P*<0.001; Supplementary Table S2 and Figure 2A). The proportions of Firmicutes, Proteobacteria and Bacteroidetes in lung bacteria were 15.447%, 70.531% and 11.779%, whereas 63.680%, 0.784% and 34.506% were in intestine bacteria, respectively. Moreover, the ratio of Firmicutes and Bacteroidetes (F/B) in lung bacteria was significantly less than those in intestine bacteria (*P* = 0.4696; Figure 2B). In addition, 30 dominant genera were found in lung and intestine bacteria (Supplementary Table S3). For instance, the relative abundances of *Lactobacillus, Romboutsia, Ruminococcaceae_UCG-014* and other genera in lung bacteria were significantly lower than those in intestine bacteria (*P*<0.001); whereas, the relative abundances of *Pseudomonas, Sphingobium, Acinetobacter* and other genera in lung microbiota were significantly higher than those in intestine bacteria (*P* < 0.001 or 0.05; and Figure 2C).

### Community structure differences between lung and intestine bacteria

**Figure 3:**
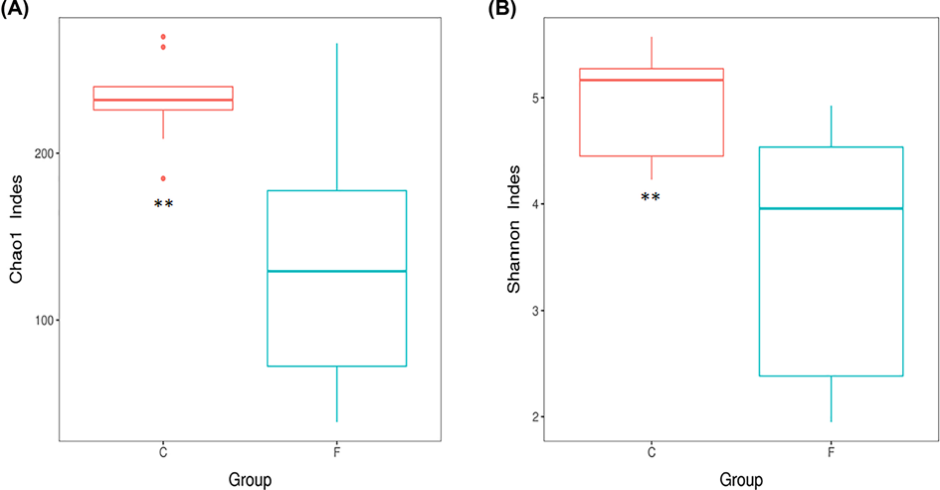
Microbial αDiversity of lung and intestine (F: lung, C: intestine) microbiotas (**A**) Chao1 index. (**B**) Shannon index. The results were showed as mean ± sem. Statistical significance was denoted by **P<0.01.

**Figure 4:**
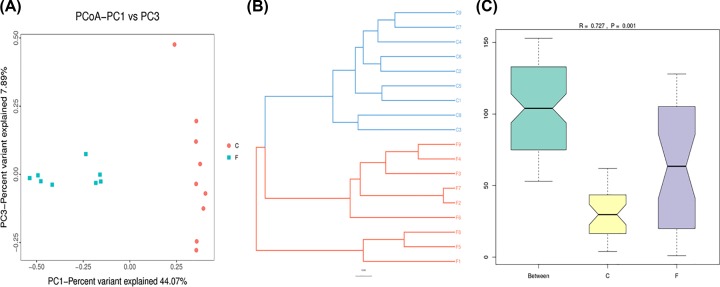
Microbial βDiversity of lung and intestine (F: lung, C: intestine) microbiotas (**A**) PCoA Plot. (**B**) UPGMA tree. (**C**) Anosim analysis. C was dispersed over an area different than that (F). There was a big difference between the two groups.

**Figure 5:**
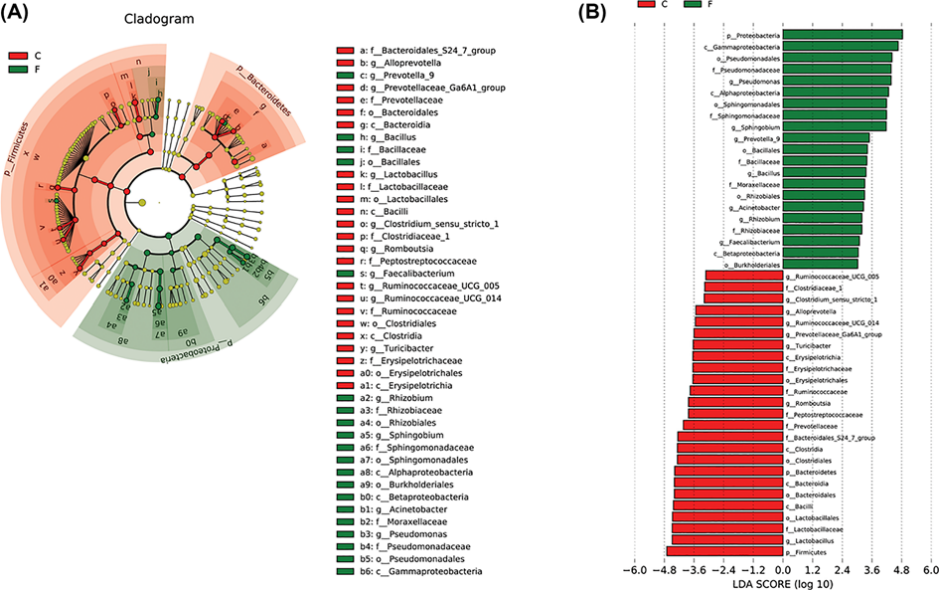
Community structure differences (F: lung, C: intestine) (**A**) Cladogram. Different color nodes represent the microbial groups that were significantly enriched in the corresponding groups and have a significant impact on the differences between groups. (**B**) Histogram. Linear discriminant analysis (LDA) effect size analysis illustrated taxa associated with C and F from the pool microbial families. Families with a LDA score >3 are shown for both data sets. The higher the LDA score, the greater the influence of species abundance on the difference.

To reveal alpha diversity of the two groups, we used the Chao1 index and Shannon index. Chao1 and Shannon indices in intestine bacteria were significantly higher than those in lung bacteria (*P*<0.001; Supplementary Table S4 and Figure 3). PCoA and UPGMA were essential parts of beta diversity and showed the differences between lung and intestine bacteria. The 18 samples were respectively clustered showing separation of lung and gut microbiome and going apart each nine samples in two groups (Figure 4A,B). In addition, the UPGMA tree showed the consistent results and statistically significant difference (*P*=0.04; Figure 4B), indicating that the 18 samples could be considered as two diverse groups. Anosim analysis showed that the differences between the two groups were significantly greater than within a group. At last, the results of Anosim analysis showed that there was significant difference in the between lung and intestine bacteria (*R*=0.727, *P*=0.01; Figure 4C). Furthermore, the LEfSe analysis indicated that all the showed bacteria played pivotal roles in lung and intestine as shown in Figure 5A,B.

### Functional differences between lung and intestine bacteria

**Figure 6:**
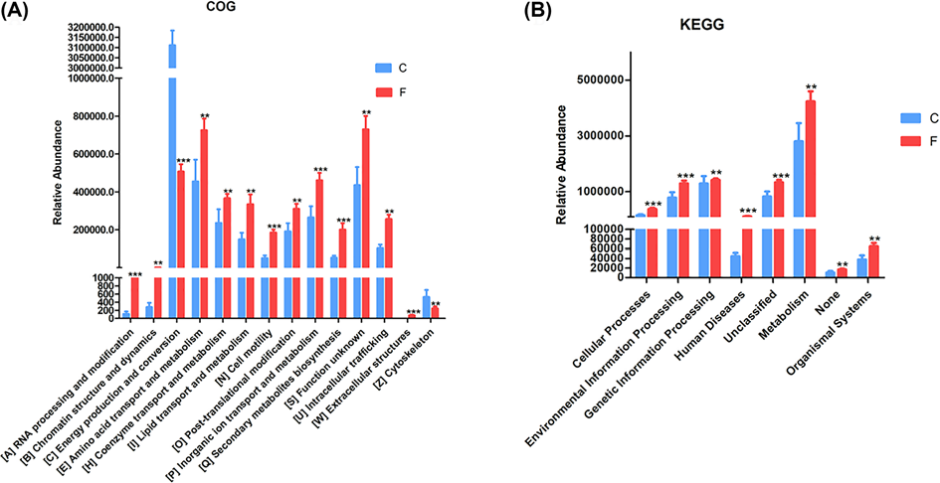
Functional differences of PICRUSt (F: lung, C: intestine) (**A**) COG functional abundance columnar distribution. (**B**) KEGG functional abundance columnar distribution.The greengene id, corresponding to each OTU was used to obtain the COG family information and KEGG Ortholog (KO) information corresponding to OTU, and the abundance and KO abundance of each COG were calculated. Then, Taxa data were used as an input to the PICRUSt software package, the results were showed as mean ± sem and filtered according to the Kruskal–Wallis *H*-test. Statistical significance was denoted by **P<0.01; ***P<0.001.

To identify functional biomarkers, functional analysis was performed to find out difference between the groups. Analysis performed includes COG and KEGG function analysis. COG function analysis displayed that the functions of all samples were much more alike to each other. The lung bacteria was diverse in their functions than intestine bacteria. We found that RNA processing and modification, chromatin structure and dynamics, amino acid transport and metabolism, co-enzyme transport and metabolism, lipid transport and metabolism, cell motility, post-translational modification, protein turnover, and chaperones, and inorganic ion transport and metabolism (*P*<0.01 or 0.001) were more dominant functions in lung bacteria communities; whereas, the intestine bacteria were associated with energy production, conversion and cytoskeleton (*P*<0.01 or 0.001; Table 1, Figure 6A). KEGG function analysis revealed that functional parameters such as environmental information processing, human diseases, metabolism, cellular processes, genetic information processing and organismal systems (*P*<0.01 or 0.001; Table 2, Figure 6B) were more dependent on lung bacteria compared with that of intestine bacteria.

**Table 1:** COG functions with the significantly different abundances between lung and intestine bacteria (mean ± SD)

Functions	*P* values	*F*	*C*
[A] RNA processing and modification	4.696 × 10^−6^	2222.444 ± 935.668	114.333 ± 57.996
[B] Chromatin structure and dynamics	1.567 × 10^−3^	3832.000 ± 2802.125	278.222 ± 109.705
[C] Energy production and conversion	5.635 × 10^−4^	507520.900 ± 116380.700	3111867.800 ± 72105.280
[E] Amino acid transport and metabolism	1.808 × 10^−3^	725406.100 ± 184101.600	455830.100 ± 114101.500
[H] Coenzyme transport and metabolism	1.109 × 10^−3^	366654.200 ± 67664.180	236208.300 ± 71832.680
[I] Lipid transport and metabolism	2.689 × 10^−3^	335188.900 ± 152650.700	150170.100 ± 34643.800
[N] Cell motility	4.867 × 10^−7^	185685.600 ± 47208.850	53637.110 ± 13215.880
[O] Post-translational modification, protein turnover and chaperones	1.073 × 10^−3^	310446.200 ± 78506.420	191884.200 ± 42675.300
[P] Inorganic ion transport and metabolism	3.348 × 10^−4^	461506.880 ± 115598.700	265146.300 ± 58938.170
[Q] Secondary metabolites biosynthesis, transport and catabolism	5.630 × 10^−4^	201262.000 ± 101231.700	55665.560 ± 11026.410
[S] Function unknown	1.533 × 10^−3^	729942.300 ± 210975.600	435578.400 ± 95691.500
[U] Intracellular trafficking, secretion and vesicular transport	1.175 × 10^−5^	256719.200 ± 70882.500	104345.700 ± 18359.500
[W] Extracellular structures	4.366 × 10^−5^	77.333 ± 41.776	0.000 ± 0.000
[Z] Cytoskeleton	1.756 × 10^−3^	246.889 ± 142.219	528.667 ± 175.069

**Table 2:** KEGG functions with significantly different abundances between lung and intestine bacteria (mean ± SD)

Functions	*P* values	*F*	*C*
Cellular processes	8.167 × 10^−5^	377599.70 ± 122497.10	154778.70 ± 36012.70
Environmental information processing	5.508 × 10^−4^	1298510.00 ± 299287.20	781358.40 ± 201523.80
Genetic information processing	2.276 × 10^−3^	1422247.00 ± 164000.00	1289690.00 ± 271235.70
Human diseases	1.782 × 10^−4^	111842.60 ± 40982.35	44617.56 ± 7160.30
Unclassified	2.023 × 10^−4^	1336734.00 ± 262654.70	821760.40 ± 187751.60
Metabolism	3.398 × 10^−3^	4241240.00 ± 1070415.00	2804159.00 ± 655178.10
None	3.356 × 10^−3^	16952.560 ± 4466.745	10637.11 ± 3219.05
Organismal systems	2.246 × 10^−3^	64691.33 ± 21072.58	37821.78 ± 8694.02

## Discussion

The gut tract acts as a complex microbial community, which has been extensively found associated with a variety of chronic diseases, such as inflammatory bowel disease, Type 2 diabetes and asthma [43,44]. Although limited studies, the connection between the respiratory and gut microbiomes has recently been considered [43,45,46]. The intestinal–lung axis explains the correlation between the lung and the intestine, and intestinal flora affect pulmonary immunity through the mechanism of the intestinal–lung axis [47]. Studies focused on human beings to investigate the relationships between the intestine and lung microbiomes are limited according to ethical considerations. However, animals have been developed to test the influence of the intestine microbiome on the lung microbiome and immunity. A growing body of evidence also shows that the intimate relationship between the gastrointestinal tract and the respiratory tract, and the deterioration of chronic intestine and lung diseases show key and important characteristics, that is, the disorders and disorders of the micro-ecosystem [48]. Moreover, a study compared the bacterial communities in lung tissue biopsies, fecal samples and vaginal lavage fluids of BALB/c mice [49]. Studies have confirmed that IL-25 induces the migration of ILC2 from the intestine to the lungs, that is, it participates in “type 2 immunity” through the intestinal–lung collecting axis of ILC2 [50]. Even, the clinical trials have shown the relationships between the intestine microbiome and the lung related in chronic respiratory diseases (CRD) patients [45,46], which revealed a significant proportion of bacteria increasing in the gut were also increasing in the respiratory tract. Furthermore, there is an authentic link between nutrition and the microbial lung community [45]. All of these evidences demonstrate that intestine microbiota and nutritional factors are related to the lung bacteria. In general, these studies reveal that the intestine microbiota have an effect on the maintenance and inflammation of the respiratory bacteria, as well as lung immunity. Further studies related to the structures and functional analysis in intestine and lung microbiotas are likely to yield important insights into the dynamics and homeostasis of microbiomes, consequently yielding a better understanding of gut dysbiosis may relate to the pathogenesis of CRD.

In the present study, we found that the intestine bacteria of rats mainly consisted of the phyla Firmicutes and Bacteroides, the genus *Lactobacillus* that plays an important role in intestine bacteria. On the other hand, the lung bacteria was mainly composed of the phylum Proteobacteria and the genus *Pseudomonas*. From microecological point of view, our study exhibited that intestine bacteria was more abundant and diverse than lung bacteria.

Firmicutes and Bacteroides play leading role in intestine bacteria and were found associated with obesity [51]. Bradley and Pollard revealed that Proteobacteria explained the variability of human intestine bacteria [52]. *Lactobacillus* was proved to improve colitis [53]. *Pseudomonas* could promote intestinal epithelial cell apoptosis [54]. Therefore, the structure diversity between the lung and intestine bacteria may be one of the reasons for their diverse functions in lung and intestine. Detailed study on the functions of these important bacterial communities may be of guiding significance in the prevention and treatment of lung and intestinal diseases.

Our results were consistent with previous studies. For instance, Robertson et al. 2017 also considered that intestine microbiota was mainly composed of Firmicutes and Bacteroides, and even used Firmicutes/Bacteroides as a reference for judging the balance of intestine bacteria [55]. Singh et al. also elucidated that Proteobacteria was the major phylum in lung bacteria [56]. At present, there is no common criterion to judge the balance of lung bacteria. Previously, we used TCM to interfere with flora and immune disorder in model rats, and found that the balance in lung and intestine bacteria of model rats was improved [57].

Our study indicated that intestine bacteria were more concentrated in energy production and conversion and cytoskeleton, compared with lung bacteria. The possible reason is that intestine bacteria has the digestive function, enriches with large number of cells and requires more bacteria enrichment [58]. Intestine bacteria produced energy-related metabolites, such as glucose and mannose [59]. The lung bacteria was more enriched in RNA processing and modification, chromatin structure and dynamics, amino acid transport and metabolism, co-enzyme transport and metabolism, lipid transport and metabolism, cell motility, post-translational modification, protein turnover, and chaperones, and inorganic transport and metabolism. They may be the lungs that are associated with gas exchange, which requires immune defense and produces a large number of antibodies. Therefore, the functions of lung microbiota were more concentrated in protein processing and synthesis [60,61] and immune defense-related metabolites, such as immunoglobulin A [62]. We found that lung microbiota was more dominant in functions of cellular processes, environmental information processing, genetic information processing, human diseases, metabolism and organismal systems than intestine bacterial communities. Thus, intestine bacteria has been proved as the target of many diseases nowadays [63,64].

Clinically, pulmonary diseases are often synchronous with the changes in intestine bacteria, and intestinal diseases are often synchronous with the changes in lung bacteria [65,66]. Understanding the structure and function of lung and intestine bacteria may be helpful to prevent and treat pulmonary and intestinal diseases and promote the development of probiotics of lung and intestine.

In summary, we investigated the structures and functions of lung and intestine bacteria and revealed the differences in the structures and functions between lung and intestine bacteria, providing the basis for understanding the relationship between lung and intestine bacteria.

## Supplementary Material

Supplementary Tables S1-S4

## Data Availability

The data used to support this study can be made freely available.

## Abbreviations

C: cecal contents
COG: Cluster of Orthologous Group
CRD: chronic respiratory diseases
F: lung tissues
HDC: histidine decarboxylase enzyme
KEGG: Kyoto Encyclopedia of Genes and Genomes
LEfSE: LDA Effect Size
OTU: Operational Taxonomic Units
PICRUSt: Reconstruction of Unobserved States
RDP: Ribosomal Database Program
SCFAs: short-chain fatty acids
UPGMA: unweighted pair group method with arithmetic mean
