# Supplementary material for: Bacterial association and comparison between lung and intestine in rats
Source: Biosci Rep. 2020 Apr 29;40(4):BSR20191570. doi: 10.1042/BSR20191570 (PMC7189363; doi:10.1042/BSR20191570)
Supplement: Supplementary Tables S1-S4 [file BSR-2019-1570_supp.pdf]

**Supplementary Table 1: Data obtained from quality optimization of 16S rRNA sequence.**

| Samples | #PE_reads | #Nochimera | AvgLen(bp) | GC(%) | Effective (%) | The number of OTUs |
|---|---|---|---|---|---|---|
| C1 | 70416 | 49807 | 454.83 | 52.6 | 70.73 | 211 |
| C2 | 66154 | 38092 | 452.66 | 52.92 | 57.58 | 201 |
| C3 | 51878 | 30537 | 452.01 | 53.24 | 58.86 | 197 |
| C4 | 52638 | 38354 | 453.15 | 52.82 | 72.86 | 194 |
| C5 | 67902 | 49032 | 453.63 | 53.13 | 72.21 | 240 |
| C6 | 48065 | 33656 | 452.27 | 52.86 | 70.02 | 188 |
| C7 | 67081 | 48314 | 455.06 | 52.98 | 72.02 | 221 |
| C8 | 58660 | 36505 | 451.8 | 53.34 | 62.23 | 161 |
| C9 | 49488 | 39199 | 453.46 | 53.08 | 79.21 | 207 |
| F1 | 209697 | 83359 | 452.51 | 52.36 | 39.75 | 126 |
| F2 | 63854 | 47940 | 452.59 | 52.67 | 75.08 | 52 |
| F3 | 69488 | 42657 | 451.46 | 53.54 | 61.39 | 136 |
| F4 | 79277 | 50749 | 452.68 | 53.02 | 64.01 | 163 |
| F5 | 49742 | 14011 | 455.61 | 53.19 | 28.17 | 61 |
| F6 | 73497 | 55780 | 450.57 | 53.15 | 75.89 | 239 |
| F7 | 77270 | 58904 | 451.93 | 52.75 | 76.23 | 37 |
| F8 | 80863 | 19342 | 455.29 | 53.26 | 23.92 | 72 |
| F9 | 76920 | 50901 | 452.15 | 53.34 | 66.17 | 180 |

**Supplementary Table 2: The top 8 percentages at the phylum level between lung and intestine (Mean±SD) (%).**

| Taxon | N | *p* value | F | C |
|---|---|---|---|---|
| Firmicutes | 9 | $1.564 \times 10^{-22}$ | 15.447 ± 12.464 | 63.68 ± 16.196 |
| Proteobacteria | 9 | $2.272 \times 10^{-35}$ | 70.531 ± 19.440 | 0.784 ± 0.242 |
| Bacteroidetes | 9 | $1.122 \times 10^{-7}$ | 11.779 ± 9.894 | 34.506 ± 16.789 |
| Actinobacteria | 9 | 0.940 | 0.239 ± 0.196 | 0.544 ± 0.603 |
| Tenericutes | 9 | 0.960 | 0.094 ± 0.283 | 0.299 ± 0.376 |
| Spirochaetaes | 9 | 0.987 | 0.002 ± 0.007 | 0.066 ± 0.079 |
| Saccharibacteria | 9 | 0.985 | 0.033 ± 0.042 | 0.109 ± 0.112 |
| Deinococcus-Thermus | 9 | 0.984 | 0.083 ± 0.169 | 0.001 ± 0.003 |

**Supplementary Table 3: The top 30 percentages at the genus level of all groups (Mean±SD) (%).**

| Taxon | N | *p* value | F | C |
|---|---|---|---|---|
| Unclassified | 9 | $2.415 \times 10^{-4}$ | 6.77 ± 5.71 | 29.54 ± 13.37 |
| Lactobacillus | 9 | $1.068 \times 10^{-5}$ | 1.61 ± 2.44 | 29.30 ± 12.97 |
| Pseudomonas | 9 | $2.637 \times 10^{-3}$ | 23.67 ± 19.96 | 0.003 ± 0.007 |
| Escherichia-Shigella | 9 | 0.076 | 18.96 ± 29.86 | 0.09 ± 0.13 |
| Sphingobium | 9 | $4.711 \times 10^{-3}$ | 15.51 ± 14.18 | 0.005 ± 0.007 |
| Romboutsia | 9 | $1.178 \times 10^{-3}$ | 0.89 ± 1.08 | 7.95 ± 5.27 |
| Desulfovibrio | 9 | 0.103 | 4.62 ± 7.51 | 0.29 ± 0.24 |
| Turicibacter | 9 | $4.051 \times 10^{-3}$ | 0.06 ± 0.19 | 4.59 ± 4.05 |
| Bacteroides | 9 | 0.175 | 3.57 ± 5.47 | 0.93 ± 1.12 |
| Lachnospiraceae_NK4A136_group | 9 | 0.411 | 1.22 ± 2.45 | 2.14 ± 2.16 |
| Ruminococcaceae_UCG-014 | 9 | $2.914 \times 10^{-7}$ | 0.12 ± 0.17 | 3.21 ± 1.09 |
| Alloprevotella | 9 | $3.822 \times 10^{-3}$ | 0.11 ± 0.15 | 3.01 ± 2.57 |
| Prevotellaceae_Ga6A1_group | 9 | 0.030 | 0.01 ± 0.03 | 2.98 ± 3.73 |
| Blautia | 9 | 0.333 | 0.54 ± 0.63 | 2.35 ± 5.40 |
| Prevotella_9 | 9 | 0.135 | 2.48 ± 4.73 | 0.00 ± 0.00 |
| Bacillus | 9 | $5.183 \times 10^{-3}$ | 2.44 ± 2.26 | 0.003 ± 0.005 |
| Acinetobacter | 9 | $6.808 \times 10^{-5}$ | 1.83 ± 1.03 | 0.001 ± 0.003 |
| Clostridium_sensu_stricto_1 | 9 | 0.015 | 0.03 ± 0.06 | 1.67 ± 1.81 |
| Rhizobium | 9 | $3.103 \times 10^{-3}$ | 1.60 ± 1.38 | 0.00 ± 0.00 |
| [Ruminococcus]_gauvreauii_group | 9 | 0.112 | 0.15 ± 0.29 | 1.24 ± 1.92 |
| Ruminococcaceae_UCG-005 | 9 | $8.390 \times 10^{-3}$ | 0.07 ± 0.14 | 1.32 ± 1.24 |
| Lachnoclostridium | 9 | 0.067 | 0.22 ± 0.22 | 0.96 ± 1.11 |
| Ruminococcaceae_NK4A214_group | 9 | $9.030 \times 10^{-3}$ | 0.02 ± 0.12 | 0.86 ± 0.84 |
| Roseburia | 9 | $4.991 \times 10^{-2}$ | 0.67 ± 0.62 | 0.20 ± 0.24 |
| Faecalibacterium | 9 | 0.151 | 0.86 ± 1.71 | 0.001 ± 0.003 |
| Prevotella_2 | 9 | 0.112 | 0.84 ± 1.50 | 0.00 ± 0.00 |
| Christensenellaceae_R-7_group | 9 | $7.704 \times 10^{-4}$ | 0.01 ± 0.03 | 0.77 ± 0.55 |
| Prevotellaceae_NK3B31_group | 9 | $5.698\times 10^{-3}$ | 0.04 ± 0.11 | 0.72 ± 0.63 |
| Ruminococcus_1 | 9 | 0.229 | 0.26 ± 0.36 | 0.10 ± 0.13 |
| Odoribacter | 9 | 0.979 | 0.61 ± 1.09 | 0.60 ± 0.24 |

**Supplementary Table 4: The chao1 and shannon indesbetween lung and intestine(Mean±SD).**

| Taxon | N | *p* value | F | C |
|---|---|---|---|---|
| chao1 | 9 | $1.112\times 10^{-3}$ | 128.4182 ± 74.39383 | 232.4486 ± 25.83714 |
| shannon | 9 | $4.480\times 10^{-3}$ | 3.450111 ± 1.224335 | 4.907444 ± 0.5018025 |
